# Hyperreflective Material in Optical Coherence Tomography Images of Eyes with Myopic Choroidal Neovascularization May Affect the Visual Outcome

**DOI:** 10.3390/jcm9082394

**Published:** 2020-07-27

**Authors:** Yasuaki Mushiga, Sakiko Minami, Atsuro Uchida, Norihiro Nagai, Misa Suzuki, Toshihide Kurihara, Hideki Sonobe, Norimitsu Ban, Kazuhiro Watanabe, Hajime Shinoda, Kazuo Tsubota, Yoko Ozawa

**Affiliations:** 1 Department of Ophthalmology, Keio University School of Medicine, 35 Shinanomachi, Shinjuku-ku, Tokyo 160-8582, Japan; pleaseplease045@yahoo.co.jp (Y.M.); saki.love5@icloud.com (S.M.); uchidats@gmail.com (A.U.); nagai@a5.keio.jp (N.N.); misa.suzuki@suzukiganka.page (M.S.); kurihara@z8.keio.jp (T.K.); betty_vol2@ybb.ne.jp (H.S.); nban@keio.jp (N.B.); gaku047nikoniko3mickey@yahoo.co.jp (K.W.); shinoha@mac.com (H.S.); tsubota@z3.keio.jp (K.T.); 2 Laboratory of Retinal Cell Biology, Keio University School of Medicine, 35 Shinanomachi, Shinjuku-ku, Tokyo 160-8582, Japan; 3 Department of Ophthalmology, St. Luke’s International Hospital, 9-1 Akashi-cho, Chuo-ku, Tokyo 104-8560, Japan; 4 St. Luke’s International University, 9-1 Akashi-cho, Chuo-ku, Tokyo 104-8560, Japan

**Keywords:** high myopia, choroidal neovascularization, retina, visual acuity, OCT

## Abstract

The visual outcome of myopic choroidal neovascularization (CNV) after anti-vascular endothelial growth factor (anti-VEGF) therapy varies among individuals. We retrospectively analyzed the data of 24 eyes (24 patients) with treatment-naïve myopic CNV who underwent anti-VEGF monotherapy following a pro-re-nata regimen at the Division of Medical Retina Clinic, Department of Ophthalmology, Keio University Hospital between May 2014 and December 2017. The mean age was 70.6 ± 2.1 years, and 16 (66.7%) patients were female. Overall, the mean best-corrected visual acuity (BCVA) improved (*p* = 0.034), and the mean height of the hyperreflective material (HRM), involving the CNV lesion recorded by optical coherence tomography, decreased (*p* < 0.01) 12 months after the initial treatment. Fifteen eyes (62.5%) achieved a BCVA of better than 0.10 in LogMAR at 12 months; they had a better BCVA (*p* = 0.015) and lower HRM intensity (*p* = 0.033) at baseline than the others. Remarkably, the BCVA improved (*p* < 0.05) and the HRM height (*p* < 0.01) decreased only in eyes with a final BCVA better than 0.10 as early as 1 month after the initial treatment, which was still present at 12 months. The HRM height and intensity, not only the BCVA, would be valuable in evaluating the prognosis of myopic CNV after anti-VEGF therapy, although further study is required.

## 1. Introduction

The incidence of myopia is rising in East Asia [1], the US [2], and Europe [3], as reported in a 2015 News and Comments article in *Nature* titled “The Myopia Boom”. Myopic choroidal neovascularization (CNV), which causes untreatable vision loss in highly myopic eyes [4], has become a leading cause of blindness [5,6,7] and comprises a worldwide social issue. The application of anti-vascular endothelial growth factor (anti-VEGF) therapy has improved the overall visual outcome of myopic CNV [1,8,9,10,11,12,13,14]. However, the outcome varies according to the individual. Elucidating the background of the differences in outcome could help delineate the pathogenesis and help better inform the patients when obtaining consent for treatment.

The diagnosis of myopic CNV is based on the clinical findings from fundus examinations, angiographies, and optical coherence tomography (OCT) [1,12]. Because it is non-invasive and requires a short recording time, OCT is also widely utilized for the follow-up of myopic CNV and of other macular diseases in the daily clinic. In general, OCT images are used to evaluate intraretinal conditions, including retinal edema [15,16] and disruptions of the ellipsoid zone (EZ) and external limiting membrane (ELM), which may reflect photoreceptor disorganization and affect the visual outcome [17,18,19], as well as subretinal lesions and/or fluid [19] in macular diseases. In OCT images, myopic CNV presents as a highly reflective area contiguous above the retinal pigment epithelium (RPE) (type 2 CNV [1]), usually with minimal fluid exudation [1]. Therefore, damage to the outer retina, such as disruptions of the EZ and ELM, is observed in most eyes with myopic CNV [20], and the findings may not help predict variations in the treatment outcome. In addition, intra- and/or sub-retinal fluid exudation may not be useful as a biomarker for disease activity. Previous reports have proposed the fuzzy borders of a highly reflective material (HRM), involving CNV, as an indicator of exudative changes in CNV [21,22]; however, it is not a quantitative marker.

In this study, we evaluated the OCT findings over time during anti-VEGF therapy in real-world clinical practice and compared the OCT findings of eyes with or without a good final visual acuity after treatment. The results could help estimate its prognosis, which could be valuable for better informing the patients when securing their consent before treatment.

## 2. Experimental Section

The study adhered to the tenets of the Declaration of Helsinki, was approved by the Ethics Committee of the Keio University School of Medicine (Tokyo, Japan; 2010003), and was registered as UMIN000007649. Informed consent was obtained from all the subjects.

### 2.1. Subjects

The medical charts of 24 eyes of 24 patients who were diagnosed with myopic CNV and unilaterally treated with either intravitreal ranibizumab or aflibercept monotherapy at the Medical Retina Division Clinic of the Department of Ophthalmology, Keio University Hospital between May 2014 and December 2017 were retrospectively reviewed. Fluorescein angiography (FA) and indocyanine green angiography (IA) were performed for diagnosis using a retinal camera (TRC50DX, Topcon, Tokyo, Japan). All the patients attended the clinic for at least 12 months, during which time they were treated with anti-VEGF monotherapies alone. Patients who had a macular hole, rhegmatogenous retinal detachment, myopic macular retinoschisis, dense opacity of the intermediate optic media, severe glaucoma, and those who had undergone eye surgeries other than cataract surgery before the initial treatment or any eye surgery during the 12 months of follow-up were excluded (Supplementary Figure S1).

### 2.2. Ocular Examinations

All the patients underwent best-corrected visual acuity (BCVA) measurements with refraction test, slit-lamp examination, and binocular indirect ophthalmoscopy after pupil dilation with 0.5% tropicamide. These examinations were performed at every follow-up visit.

### 2.3. OCT

Horizontal and vertical OCT images across the fovea were recorded at every follow-up visit using an OCT instrument (Spectralis, Heidelberg Engineering, Dossenheim, Germany) with the OCT section high resolution mode (30°, average of a minimum of 50 frames acquired using a retinal tracking system). The repeat mode was applied at the time of the follow-up visits to obtain OCT images at the exact same location. The height of the HRM at the fovea was defined as the distance between the bottom and top of the lesion. The HRM intensity was defined by measuring the intensity values of the lesion, vitreous cavity, and the RPE using ImageJ (https://imagej.nih.gov/ij/) (range, 0–255), and evaluated by the following calculation formula: HRM intensity = (HRM intensity—vitreous intensity)/(RPE intensity—vitreous intensity).

### 2.4. Intravitreal Injections

Either ranibizumab (0.5 mg (0.05 mL)) or aflibercept (2 mg (0.05 mL)) monotherapy was administered. The drug was injected intravitreally under sterile conditions via the pars plana once, and then as needed (pro-re-nata). If the eyes had any intra- and/or subretinal fluid and hemorrhage in the fundus or new symptoms at the time of the visits, they were re-treated at intervals longer than 1 month. If the lesion and/or symptoms improved but remained, the lesion was considered to be responding, and the injections were repeated until there was no further improvement. When no fluid or hemorrhage was detected for more than 2 months, the follow-up interval was extended from 1 month to up to 2 months.

### 2.5. Statistical Analyses

All the results are expressed as the mean ± the standard error. Commercially available software (SPSS version 25.0, IBM Corporation, Armonk, NY, USA) was used for the statistical analysis. A generalized analysis of variance (ANOVA), the Mann–Whitney *U*-test, the chi-square test, the Pearson correlation coefficient, and univariate and multivariate linear regression analyses were used. Differences were considered statistically significant at *p* < 0.05.

## 3. Results

### 3.1. Participants’ Characteristics

**Figure 1:**
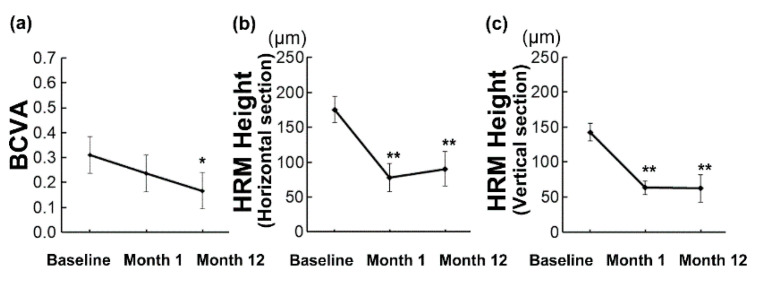
Changes in the best-corrected visual acuity (BCVA) and height of hyperreflective material (HRM) in the optical coherence tomography images over time. (**a**–**c**) Mean ± standard errors are shown. Generalized analysis of variance was performed. Overall, the mean BCVA improved at 12 months (**a**), while the mean HRM heights improved as early as 1 month after the initial treatment and the effect was still present at 12 months (**b**,**c**). * *p* < 0.05, ** *p* < 0.01.

The mean age of the 24 patients who had unilateral treatment naive myopic CNV and were treated with anti-VEGF monotherapy in the current study was 70.6 ± 2.1 years (range, 50–83 years) (Table 1). Sixteen (66.7%) patients were female, and eight (33.3%) patients were male. The mean initial BCVA was 0.31 ± 0.07 (range, −0.08–1.3) in LogMAR (Table 1, Figure 1a), and the mean intraocular pressure (IOP) was 13.5 ± 0.7 (range, 7–20) mmHg. Seventeen eyes (70.8%) had a subfoveal CNV, and 7 eyes (29.2%) had a juxtafoveal CNV. (Table 1). The HRM was recorded in 22 eyes (91.7%) in horizontal sections and in 19 eyes (79.2%) in vertical sections of the initial OCT images in both directions across the fovea (Table 1). There was a correlation in the HRM heights (*r* = 0.662, *p* = 0.004) and a trend of correlation in the HRM intensities (*r* = 0.436, *p* = 0.080), between the values measured in the horizontal and vertical OCT sections (Supplementary Figure S2, Pearson correlation coefficient).

The mean injection number was 2.16 ± 1.40 (range, 1–7) during the 12 months. Six eyes (25%) received aflibercept monotherapy, and 18 eyes (75%) received ranibizumab monotherapy (Supplementary Table S1). There were no differences in the BCVA at month 12 (Mann–Whitney *U*-test, *p* = 0.16, Supplementary Table S1) and injection number during the 12 months (Mann–Whitney *U*-test, *p* = 0.08, Supplementary Table S1) according to the therapy received.

### 3.2. Overall Changes in BCVA and HRM Height during 12 Months

Overall, the mean BCVA improved at 12 months (0.17 ± 0.07 (range, −0.08–1.2), *p* = 0.034, Figure 1a), while there was no change at 1 month (0.24 ± 0.07 (range, −0.08–1.5), *p* = 0.547) after the initial treatment. For HRM analyses, we included the eyes in which HRM was initially detected and analyzed in each section direction separately. The eyes in which the lesion disappeared afterwards, although initially detected, were included in the height analyses over time, and the height was counted as 0 when it disappeared. The respective mean HRM heights at baseline, 1 month, and 12 months after initial treatment in the horizontal sections were 175.4 ± 18.9 (range, 49–452, 77.7 ± 20.0 (range, 0–420), and 90.4 ± 25.1 (range, 0–465) μm, and the respective values in the vertical sections were 142.7 ± 12.8 (range, 36–229, 63.1 ± 9.7 (range, 0–145), and 62.4 ± 19.5 (range, 0–358) μm. The mean values were significantly reduced 1 month after the initial treatment (both in the horizontal and vertical sections; *p* < 0.001), and the effect was still present at 12 months (both *p* < 0.001) (Figure 1b,c).

### 3.3. Characteristics of Initial HRM in Eyes with or without BCVA ≤ 0.10 at 12 Months After the Initial Treatment

Subsequently, we divided the patients into two groups according to the BCVA at 12 months. Fifteen eyes (62.5%) had a better BCVA than 0.10 in LogMAR (BCVA ≤ 0.10, and 20/25 in Snellen Chart and 0.8 in Landolt C Chart) at 12 months. The patients who had a better BCVA than 0.10 in LogMAR (BCVA ≤ 0.10) at 12 months also had a better BCVA at baseline (0.16 ± 0.04, Table 2; *p* = 0.015) compared with those who had a worse BCVA than 0.10 at 12 months (BCVA > 0.10, 0.56 ± 0.15 at baseline, Table 2). The initial HRM height did not differ between the groups (Table 2). However, the initial HRM intensity, which may reflect differences in the tissue components of the CNV-related lesion, was significantly lower in the group with a better BCVA at 12 months in the horizontal OCT sections (Table 2; *p* = 0.033). There was a similar trend in the vertical sections (Table 2; *p* = 0.075). The respective injection numbers of the final BCVA better and worse than 0.10 in LogMAR were 2.00 ± 3.73 and 2.27 ± 4.41, and the difference was not significant (Table 2; *p* = 0.861).

**Figure 2:**
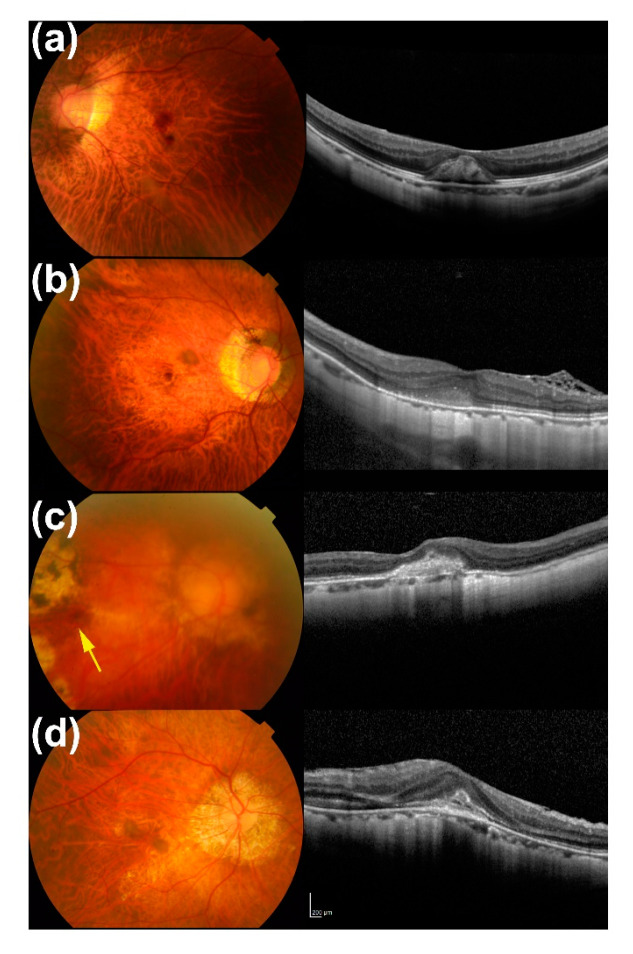
Representative initial fundus photographs and optical coherence tomography (OCT) images of the patients with or without low (**a**,**b**) and high (**c**,**d**) hyperreflective material (HRM) intensity. (**a**) A 50-year-old man whose eye had a low HRM intensity (45.3); the respective best-corrected visual acuities (BCVAs) at baseline, 1 month, and 12 months were 0.30, 0.15, and 0.05 in LogMAR. (**b**) A 64-year-old man whose eye had a low HRM intensity (60.7); the respective BCVAs at baseline, 1 month, and 12 months were 0.097, 0.05, and −0.079 in LogMAR. (**c**) A 68-year-old woman whose eye had high HRM intensity (105.4); the respective BCVAs at baseline, 1 month, and 12 months were 1.30, 1.00, and 1.22 in LogMAR. Arrow in the fundus photograph shows the lesion. (**d**) A 73-year-old man whose eye had a high HRM intensity (91.0); the respective BCVAs at baseline, 1 month, and 12 months were 0.222, 0.301, and 0.155 in LogMAR.

The representative initial OCT images and fundus photographs of the patients with a better final BCVA and low initial HRM intensity (Figure 2a,b), and worse final BCVA and high initial HRM intensity (Figure 2c,d) are shown.

### 3.4. Differences in the Clinical Courses of Eyes with or without BCVA ≤ 0.10 at 12 Months After Initial Treatment

**Figure 3:**
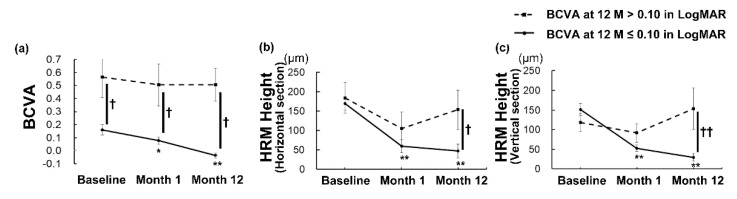
Changes in the best-corrected visual acuity (BCVA) and height of the hyperreflective material (HRM) over time in the eyes with or without a visual outcome better than 0.10 in LogMAR at month 12. (**a**–**c**) Mean ± standard errors are shown. Generalized analysis of variance was performed to compare the values at each time point and at baseline within each group (* *p* < 0.05, ** *p* < 0.01), and the Mann–Whitney *U*-test was used to compare the values between the groups (^†^ *p* < 0.05, ^††^ *p* < 0.01). Mean BCVA (a) and mean HRM heights (b,c) improved as early as 1 month after initial treatment, and the effect was still present at 12 months in the eyes that achieved a BCVA better than 0.10 in LogMAR 12 months after the initial treatment. The improvement was not observed in the eyes that had a BCVA worse than 0.10 in LogMAR at 12 months.

In the group with a better BCVA at 12 months, the improvement in BCVA compared with the baseline was already significant 1 month after the initial treatment (*p* < 0.05) and was still present at 12 months (*p* < 0.01) (Figure 3a). However, it was not improved at 1 and 12 months after initial treatment in the group with a worse final BCVA (Figure 3a). The between-group differences in BCVA were significant at each time point (*p* < 0.01).

Although the mean HRM height did not differ between the groups at baseline, the mean HRM height decreased as early as at 1 month, and the effect was still present at 12 months compared with the initial height only in the group with a final BCVA better than 0.10, both in the horizontal and vertical OCT images (Figure 3b, c; *p* < 0.001 for all). There were no differences between the values at baseline, 1 month, and 12 months after initial treatment in the group with a final BCVA worse than 0.10 (Figure 3b, c; *p* > 0.05 for all). The HRM height was significantly smaller in the group with a better final BCVA than in the other group at 12 months both in the horizontal (Figure 3b, *p* < 0.05) and vertical sections (Figure 3c, *p* < 0.01). The reduction rate of the HRM height at 1 month from the baseline was significantly greater in the group with a better final BCVA in the vertical sections (64.6 ± 6.4 (23.1 to 100) %) than in the other group (10.8 ± 23.1 (−58.3 to 100) %) (Supplementary Table S2; *p* < 0.01). 

### 3.5. Impact of Initial BCVA and HRM in Predicting BCVA at 12 Months After the Initial Treatment

A univariate linear regression analysis showed that the baseline BCVA was significantly correlated with the BCVA at month 12 (coefficient, 0.673; 95% Confidence Interval, 0.354–0.991, *p* < 0.001) (Table 3), and the adjusted R-squared was 0.442. Interestingly, a multivariate linear regression analysis including both the baseline BCVA and HRM intensity in the horizontal section showed an adjusted R-squared of 0.502 (Table 4), suggesting that the HRM intensity itself may not predict the final BCVA; however, adding the information of HRM intensity may strengthen the prediction of BCVA at month 12.

## 4. Discussion

Overall, the BCVA improved and the HRM decreased by intravitreal anti-VEGF therapy. Fifteen eyes (62.5%) achieved a BCVA better than 0.10 in LogMAR at 12 months after the initial treatment, and these eyes had a better BCVA and lower HRM intensity at the baseline. Remarkably, the BCVA improved only in eyes that achieved a better BCVA at 12 months, and the improvement was already significant at 1 month after the initial treatment. In the same group, the HRM height decreased as early as 1 month after initial treatment, and the effect was still present at 12 months. In contrast, the eyes that had a final BCVA worse than 0.10 had no improvement in BCVA or HRM height both at 1 and 12 months after the initial treatment.

Among the 24 patients, 66.7% were female, which is consistent with previously reported samples. The prevalence of high myopia in a Japanese population-based cohort was 3.8% in males and 5.9% in females, and thus was more prevalent among females than among males [23]. Females comprised 75% in a randomized clinical trial of anti-VEGF therapy for myopic CNV called Radiance [8], 61% in the Taiwanese National Health Insurance claims database of patients with CNV [24], and 77% [25] and 80% [22] in two independent Korean hospital-based studies. Thus, high myopia is more often observed in females, and myopic CNV would be more commonly found in females; thus, the sample composition of the current study was consistent with those previously reported.

The initial BCVA was better in the group with a good final BCVA than in the group with a worse final BCVA (not to be confused with a decrease in BCVA after treatment), consistent with previous reports of visual outcome being related to the initial BCVA [25]. The concept that a better initial BCVA leads to a better final BCVA is supported by between-study comparisons; the respective mean BCVA before and 12 months after anti-VEGF treatment in a historical study conducted between 2007 and 2010 was 0.57 and 0.40 in LogMAR [25]; in another conducted between 2009 and 2015, it was 0.74 and 0.65 in LogMAR [22]; and both studies reported relatively worse initial and final BCVAs. In contrast, the current study was conducted between 2014 and 2017 with an initial BCVA of 0.31 in LogMAR and a relatively better mean BCVA, resulting in a good visual outcome of 0.17 in LogMAR in the overall average. Therefore, these facts suggest that the indication of treating myopic CNV may have moved earlier, before BCVA substantially declines, and this trend could support achieving good outcomes. In the current study, the subgroups were divided by the BCVA at 12 months and not by the improvement from the baseline, considering the recent trend of starting therapy before the BCVA became declined, and still good; under this condition, the ceiling effect may affect the results. We divided the group by a BCVA of 0.10 or better, because this is the critical value for keeping a driver’s license.

Interestingly, the HRM intensity at baseline was lower in the eyes that achieved a better final BCVA at 12 months. Casalino et al. hypothesized, based on their longitudinal multimodal imaging research, that HRMs can become more defined with the reflectivity increasing over time, which may represent the maturation of the neovascular complex and evolution into scar tissue [26]. The HRM with a high intensity at baseline might have already involved mature and scar tissue in the current study; in contrast, that with a low intensity may have included more exudative changes, such as fibrin, and could be replaced by anti-VEGF therapy to achieve a better BCVA, although further study is required. Clinicians may have an impression that highly reflective material in OCT images may be related to fibrotic lesion; however, to the best of our knowledge no quantitative methods and/or data were provided previously. Intensity is a quantifiable biomarker on OCT images, and thus could be evaluated objectively, while the fuzziness of the HRM margin, which was reported by Lee et al. as a parameter of CNV activity [22], would be qualitative. It would be of value to develop a software for automatically calculating the intensity. Because most of the OCT data from the horizontal and vertical images had a similar trend, clinicians may assess either or both directions of the OCT images in the daily clinic.

Overall, the BCVA improved 12 months after the initial treatment in the current study, which is consistent with the findings of previous reports [8,9,13,27]. However, in contrast to the group with a better final BCVA at 12 months, which achieved a significant improvement in BCVA as early as at 1 month and also at 12 months compared with the baseline, the group with a worse final BCVA showed no improvement in BCVA at any of the analyzed time points. The same was true regarding the reduction in HRM height, which most likely reflected a reduction in the CNV volume and exudative changes. Taken together, eyes with myopic CNV may be divided into those that had a relatively better BCVA before treatment, responded well to the anti-VEGF therapy, and achieved a significant BCVA improvement to attain a good final BCVA; and eyes in which the BCVA had been already declined before the initial treatment, did not sufficiently respond to the therapy, and thus did not achieve BCVA improvement and had a poor final BCVA. This concept could be related to the disease stage; early stage lesions might respond well to the treatment, which leads to a better final BCVA. Initiating the treatment at early stages would be recommended.

The limitations of this study were the relatively small sample size and the retrospective design. Multiple physicians managed the patients, and treatment requirement was determined by each physician and not by the reading center, and those who used ranibizumab and aflibercept were included. The HRM may involve various components such as CNV, exudative changes (e.g., fibrin, hemorrhage, and serous exudate), and fibrotic tissue; however, there were no histological data.

In conclusion, the BCVA and HRM height overall improved with anti-VEGF therapy. However, the eyes that had a better BCVA after treatment were represented by the better BCVA and low-intensity HRM on OCT images before treatments. Those eyes already had significant BCVA improvement and HRM reduction in response to an initial single anti-VEGF drug injection, suggesting that the lesions in those eyes were composed of VEGF-responsive tissue and may not be fibrotic tissue. The initial HRM intensity did not predict the final BCVA by itself; however, it strengthened the prediction. The results may help us to understand the pathological condition of the CNV lesion and estimate the prognosis to obtain informed consent. Further analyses are warranted to evaluate the characteristics of HRM.

## Figures and Tables

**Table 1:** Baseline characteristics of the patients.

Age (years old)	70.6 ± 2.1 (50 to 83)
Sex (female [%])	16 (66.7)
Baseline BCVA (LogMAR)	0.31 ± 0.07 (−0.08 to 1.3)
Intraocular pressure (mmHg)	13.5 ± 0.7 (7 to 20)
CNV location	
Subfoveal, (eyes [%])	17 [70.8]
Juxtafoveal, (eyes [%])	7 [29.2]
HRM height (μm)	
Horizontal section	175.4 ± 18.8 (49 to 452)
Vertical section	142.7 ± 12.7 (36 to 229)
HRM intensity	
Horizontal section	65.6 ± 3.5 (35.1 to 105.4)
Vertical section	66.1 ± 3.0 (48.2 to 90.9)

Data are shown as the mean ± standard error (range). BCVA, best-corrected visual acuity; CNV, choroidal neovascularization; HRM, hyperreflective material. HRM was found and analyzed in the horizontal section of 22 and vertical section of 19 patients.

**Table 2:** Data in the patients with or without a visual outcome better than 0.10 in LogMAR at month 12.

	BCVA ≤ 0.10 in LogMAR	BCVA > 0.10 in LogMAR	*p*-Value
Eyes (%)	15 (62.5)	9 (37.5)	-
Age	68.9 ± 2.9 (53 to 86)	73.4 ± 2.7 (55 to 80)	0.299
Sex (female) (eyes [%])	10 [66.7]	6 [66.7]	1.000
Baseline BCVA	0.16 ± 0.04	0.56 ± 0.15	0.015 *
Baseline HRM height (μm)			
Horizontal section	169.2 ± 17.6 (75 to 256)	184.4 ± 39.0 (49 to 452)	0.700
Vertical section	151.4 ± 15.1 (52 to 229)	118.2 ± 22.4 (36 to 129)	0.263
Baseline HRM intensity			
Horizontal section	59.8 ± 4.0 (35.1 to 88.2)	73.9 ± 5.4 (51.3 to 105.4)	0.033 *
Vertical section	63.0 ± 2.8 (48.2 to 88.2)	75.0 ± 7.4 (54.1 to 90.8)	0.075
Injection Number	2.00 ± 3.73 (1 to 7)	2.27 ± 4.41 (1 to 4)	0.861

Data are shown as the mean ± standard error (range). Mann–Whitney *U*-tests and a chi-square test were performed. HRM, hyperreflective material. HRM was found and analyzed in the horizontal sections of 13 and the vertical sections of 14 patients among those who had a BCVA better than 0.10 in LogMAR at month 12, and in 9 and 5 patients among the others, respectively. BCVA, best-corrected visual acuity. * *p* < 0.05.

**Table 3:** Univariate linear regression analysis for BCVA at month 12.

Variable	Adjusted R^2^	Constant [95% CI]	Coefficient [95% CI]	*p*
Baseline BCVA (LogMAR)	0.442	−0.42 [−0.192, 0.108]	0.673 [0.354, 0.991]	<0.001 **
HRM intensity				
Horizontal section	0.122	−0.403 [−1.042, 0.236]	0.009 [0.000, 0.018]	0.062
Vertical section	−0.047	−0.061 [−1.032, 0.910]	0.003 [−0.011, 0.017]	0.673

Univariate linear regression analysis. BCVA, best-corrected visual acuity; HRM, hyperreflective material. ** *p* < 0.01.

**Table 4:** Multivariate linear regression analysis for BCVA at month 12.

	Unstandardized Coefficient		Standardized Coefficient	
Model	B	Standard Error	Beta	*p*
(Constant)	−0.314	0.244		0.214
Baseline BCVA (logMAR)	0.599	0.167	0.613	0.002 **
HRM intensity, Horizontal section	0.005	0.004	0.211	0.231
Total adjusted R^2^	0.502			

Multivariate linear regression analysis showing the influence of the baseline BCVA and HRM in the horizontal section on the BCVA at month 12. BCVA, best-corrected visual acuity; HRM, hyperreflective material. ** *p* < 0.01.

## Supplementary Materials

The following are available online at https://www.mdpi.com/2077-0383/9/8/2394/s1, Figure S1: A flow chart for inclusion and exclusion of the subjects, Figure S2: Correlations between the values measured in horizontal and vertical OCT sections, Table S1: Data of eyes treated with either Aflibercept or Ranibizumab monotherapy, Table S2: Reduction rate of hyperreflective material (HRM) height at 1 month after initial treatment in the patients with or without visual outcome better than 0.10 in LogMAR at month 12.
