# Supplementary material for: Hyperreflective Material in Optical Coherence Tomography Images of Eyes with Myopic Choroidal Neovascularization May Affect the Visual Outcome
Source: J Clin Med. 2020 Jul 27;9(8):2394. doi: 10.3390/jcm9082394 (PMC7466026; doi:10.3390/jcm9082394)
Supplement: Supplementary file 1 [file jcm-09-02394-s001.pdf]

Supplementary Table S1. Data of eyes treated with either Aflibercept or Ranibizumab monotherapy.

	**Aflibercept**	**Ranibizumab**	***p*-Value**
Eyes (%)	6 (25)	18 (75)	–
Sex (female [%])	3 (50)	13 (72)	0.362
BCVA at baseline (LogMAR)	0.148 ± 0.0624	0.366 ± 0.0932	0.280
BCVA at month 12 (LogMAR)	−0.016 ± 0.0309	0.228 ± 0.0923	0.160
Injection Number	3.17 ± 0.833	1.83 ± 0.232	0.080

Data are shown in mean ± standard error (range). Chi-square test and Mann-Whitney *U*-tests were performed. BCVA, best-corrected visual acuity. * $p < 0.05$.

Supplementary Table S2. Reduction rate of hyperreflective material (HRM) height at 1 month after initial treatment in the patients with or without visual outcome better than 0.10 in LogMAR at month 12.

Reduction in HRM height (%)	BCVA < 0.10 in LogMAR	BCVA ≥ 0.10 in LogMAR	*p*-Value
Horizontal section	61.9 ± 9.2 (−9.3 to 100.0)	42.9 ± 12.3 (6.1 to 100.0)	0.222
Vertical section	64.6 ± 6.4 (23.1 to 100.0)	10.8 ± 23.1 (−58.3 to 100.0)	<0.01 **

Data are shown in mean ± standard error (range). Mann-Whitney *U*-tests were performed. HRM, Hyperreflective material. HRM was found and analyzed in the horizontal sections of 13 and vertical sections of 14 patients among those who had BCVA better than 0.10 in LogMAR at month 12, and 9 and 5 patients among the others, respectively. ** $p < 0.05$.

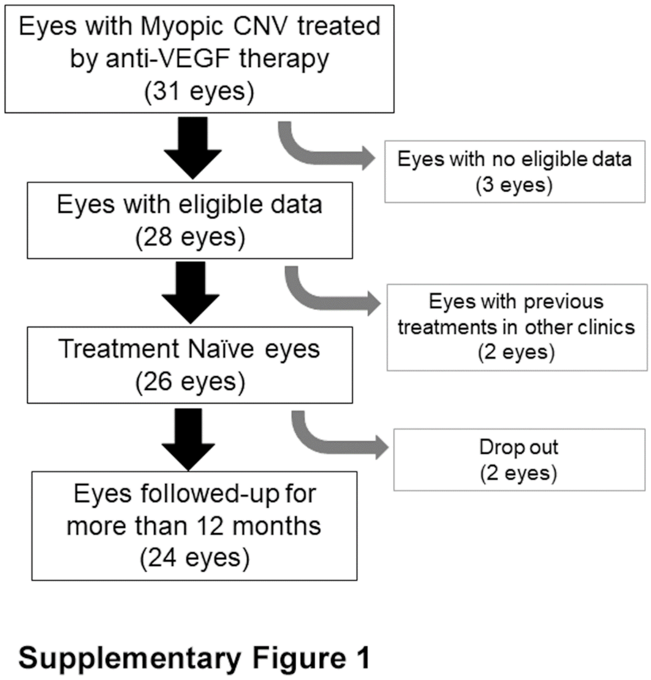

Supplementary Figure S1. A flow chart for inclusion and exclusion of the subjects

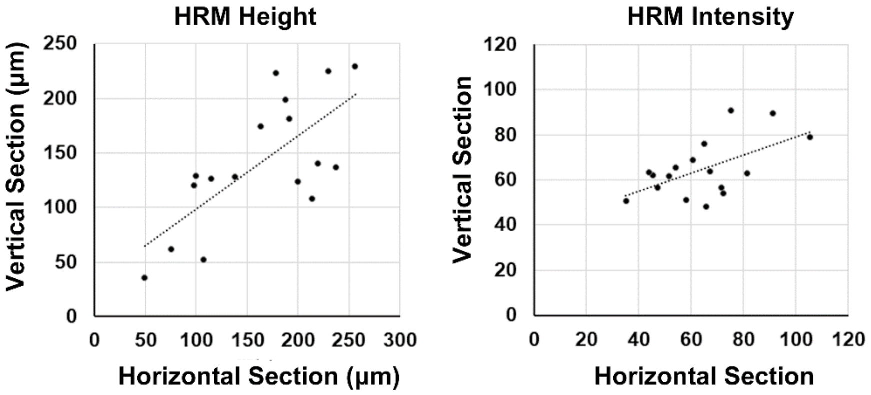

Supplementary Figure 2

Supplementary Figure 2. Correlations between the values measured in horizontal and vertical OCT sections

There was a correlation in HRM heights (r=0.662, p=0.004) and a trend of correlation in HRM intensities (r=0.436 p=0.080), both between the values measured in horizontal and vertical OCT sections (Pearson correlation coefficient). HRM, hyperreflective material.
